# Anesthetic management for cesarean delivery in a woman with congenital atlantoaxial dislocation and Chiari type I anomaly: a case report and literature review

**DOI:** 10.1186/s12884-021-03751-3

**Published:** 2021-04-01

**Authors:** Yuyan Nie, Weimin Zhou, Shaoqiang Huang

**Affiliations:** 1 Department of Anesthesiology, The Obstetrics and Gynecology Hospital of Fudan University, Shanghai, China; 2 Department of Anesthesiology, Anyang Maternity and Child Care Center, Anyang City, Henan Province China

**Keywords:** Anesthetic management, Congenital atlantoaxial dislocation Arnold–Chiari malformation, Cesarean section, Syringomyelia

## Abstract

**Background:**

The preferable choice of anesthesia for the patients with congenital atlantoaxial dislocation (CAAD) and type I Arnold Chiari malformations (ACM-I) has been a very confusing issue in clinical practice. We describe the successful administration of combined spinal-epidural anesthesia for a woman with CAAD and ACM-1 accompanied by syringomyelia.

**Case presentation:**

Our case report presents the successful management of a challenging obstetric patient with CAAD and ACM-1 accompanied by syringomyelia. She had high risks of difficult airway and aspiration. The injection of bolus drugs through the spinal or epidural needle may worsen the previous neurological complications. The patient was well evaluated with a multidisciplinary technique before surgery and the anesthesia was provided by a skilled anesthesiologist with slow spinal injection.

**Conclusions:**

An interdisciplinary team approach is needed to weigh risks and benefits for patients with CAAD and ACM-1 undergoing cesarean delivery. Therefore, an individual anesthetic plan should be made basing on the available anesthetic equipments and physicians’ clinical experience on anesthetic techniques.

## Background

Congenital atlantoaxial dislocation (CAAD) is a type of joint dysfunction and/or nerve compression pathologic disorder. It results from the congenital cervical spine cord malformation in which the articular surface of the atlas and axis (the first and second cervical vertebrae) is not aligned properly [1]. This congenital malformation of the cervical spinal cord can result in the downward displacement of brain tissue which is called Arnold Chiari malformations (ACMs). The cerebellum, pons, and medulla oblongata herniate through the foramen magnum in patients with ACM. There are four types of ACMs. Type I (ACM-I) is the most common one. The cerebellar tonsils and lower part of the medulla oblongata prolapse through the foramen magnum without displacement of the fourth ventricle in type I [2]. The prevalence rate of ACM-I in the general population is up to from 0.56 to 0.77% [3]. However, ACM caused by CAAD is rare. Syringomyelia could be likely to develop due to craniospinal pressure dissociation caused by brain tissue displacement. About 25% ~ 30% of ACM-I patients are accompanied with syringomyelia [3]. The signs and symptoms in patients with CAAD depend on the site and extent of compression at cervicomedullary junction. Its clinical symptoms are varied, such as headache, neck and shoulder pain, sensorimotor abnormalities of limbs, autonomic nervous dysfunction and so on.

The anesthetic management of cesarean section in the parturient with CAAD and ACM accompanied by syringomyelia presents many challenges to anesthetic staff. The choice for the mode of anesthesia is crucial. When the neuraxial technique is chosen, rapid bolus injections into spinal space and accidental dural puncture may lead to the significant fluctuations in CSF pressure. This would be likely to increase the degree of cerebellar herniation and enlarge the syrinx. However, the limited neck movement and mouth opening induced by CAAD would also expose the patient to a high risk of difficult intubation when general anesthesia is selected. We present the anesthesia administration and anesthetic management for cesarean section in a woman with CAAD and ACM accompanied by syringomyelia.

## Case presentation

This case report obtained written permission from the patient to publish. A 32-year-old, gravida 2 para 0 parturient, height 158 cm, weight 69 kg was presented at 35 + 5 weeks of gestational age because of hypertension with blood pressure 171/113 mmHg. Upon admission, the patient was diagnosed with severe preeclampsia and treated with intravenous magnesium sulfate and oral nifedipine. A cesarean section was scheduled. Past medical history: at age 16, she complained of unsteady walking and exhibited mild ataxia. She had been diagnosed with multiple cervical deformities, congenital atlantoaxial joint subluxation, syringomyelia, cerebellar tonsillar hernia, congenital basilar invagination. Neck traction therapy had been undergone at that time. But the patient continued to have symptoms such as nystagmus, hypohidrosis on the left side of the face, progressive dysphagia, paresthesias and weakness of the right extremities accompanied by hypalgesia, thermohypoesthesia, ataxia, and visual disturbance.

**Fig. 1:**
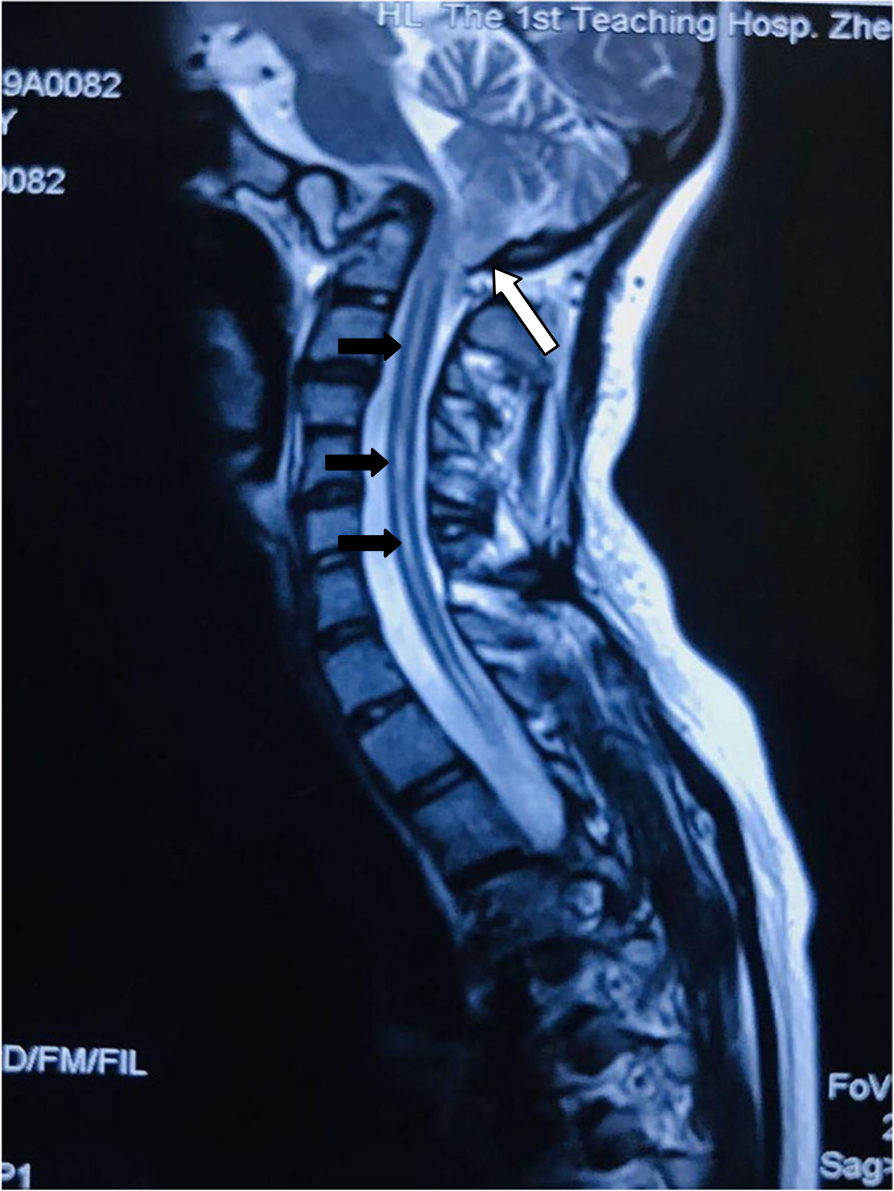
T2-weighted sagittal MRI of the upper spinal cord and brainstem in a pregnant woman with Congenital Atlantoaxial Dislocation and Chiari type I anomaly accompanied by syringomyelia. The syrinx is indicated from C2 to T 9 (black arrows). The cerebellar tonsil herniates through the foramen magnum (white arrow)

**Fig. 2:**
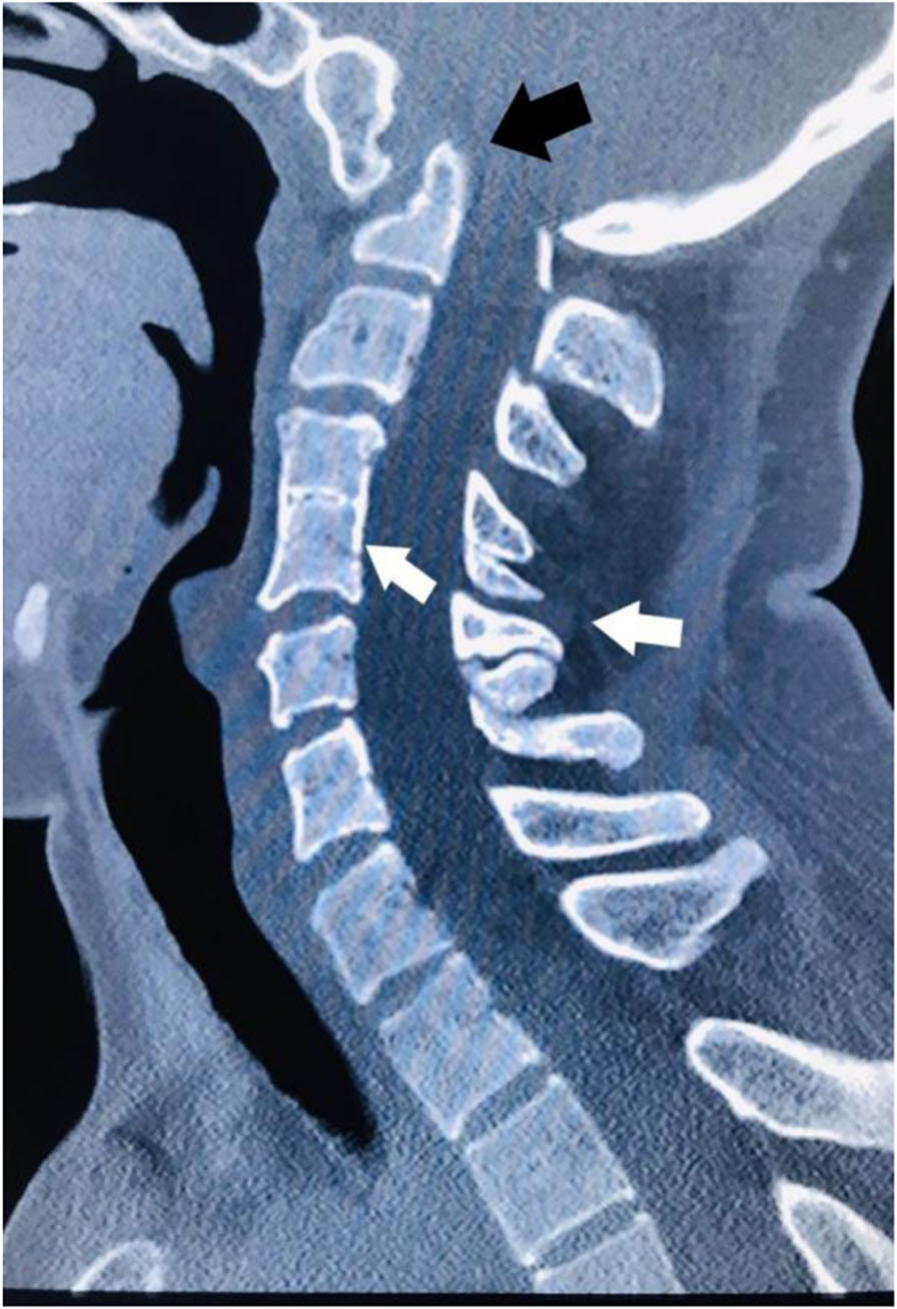
Computed Tomography demonstrates that the odontoid of the axis protrudes into the skull (black arrow). Multiple cervical vertebrae deformity and fusion are also seen (white arrows)

Neurological examination conducted by the neurologist showed that (1) the pyramidal tract was impaired: a variety of pathological reflexs of the right side were all positive such as Babinski Reflex, Brudzinski sign, Oppenheim′s sign and Gordon sign; (2) The lower central nervous system of the right spinal cord was damaged: hyperreflexia on the right side such as hoffmann’s sign (C7-T1), radioperiosteal reflex (C5-C8), biceps reflex (C5–6) and triceps reflex (C6-C7). Recent MRI imaging of the brain and spine demonstrated that the anterior atlantovertebral arch was fused adjacent to the slope. The vertebral arch of the atlas was not shown. The odontoid process was significantly displaced backward and upward, protruding through the skull base. Medulla oblongata and cervicomedullary junction was obviously compressed and backward moved. The cerebellar tonsils herniated through the foramen magnum. Syringomyelia was seen. There were abnormal signals of syrinx from Cervical 2 to Thoracic 9 vertebral body level, which was assumed as syringomyelia (Fig. 1). CT demonstrated lateral curvature of cervical vertebra bodies. The atlantoaxial joint was asymmetric. The boundary between atlas and skull base was not clear. C4, 5 space narrowed and fused partly. The spinous processes of C3, 4, 5 were irregular in shape and not fused thoroughly. The physiological curve of the lumbar spine becomes straight (Fig. 2). ACM-1 malformation was diagnosed by the neurologist according to the patient’s imaging findings and symptoms.

The preoperative anesthetic evaluation: Severe cervical deformity (short neck, cervical spinal scoliosis), limited neck movement, a Mallampati grade III airway, limited mouth opening with an interdental distance of 4 cm, thyromental distance 4 cm and bite upper-lip test grade 1 were demonstrated. Difficult intubation was supposed. The risk of aspiration should be concerned because the patient continuously suffered progressive dysphagia. On palpation she exhibited a degree of back oedema because of severe preeclampsia, but her vertebral spines were easily identified. Furthermore, no lumbar syringomyelia was found in MRI. Baseline laboratory values were hemoglobin 11.6 g/dl and platelets 208 × 10^9^/l. Considering all the above factors, the anesthetic plan in this case was carefully made using a multidisciplinary approach and a combined spinal and epidural anesthesia was eventually determined. General anesthetic techniques with fiberoptic intubation were also considered as an alternative option if the neuraxial technique failed. The patient and her family were informed about the anesthetic plan and risks of the procedure. Consent was obtained, and she was transferred to the operating room.

The patient received standard monitoring with ECG, noninvasive blood pressure (BP) and oxygen saturation. Oxygenation was administered via a mask at 8:50 am(5 L/min). ECG showed sinus rhythm. The patient’s basic vital signs were BP 145/90 mmHg, heart rate (HR) 82 bpm, SPO2 100%, and respiratory rate (RR) 20 breaths/ minute. Fiberoptic bronchoscopy preloaded with a 7.0 endotracheal tube and suction device were also prepared for general anesthesia. The neuraxial procedure was performed on the right lateral decubitus at L2–3 with the needle-through-needle technique [Disposable anesthesia puncture kit (A 28-G pencil point spinal needle, a 17-G Tuohy epidural needle, a flexible 19-G spring wound closed-tip catheter), Zhejiang Haisheng medical Device Co. LTD; Shaoxing, China]. An epidural catheter was threaded 5 cm cephalally into the epidural space. The procedure was performed by an experienced and trained anesthesiologist. Ropivacaine (Nalopine, 10 mg/ml, AstraZeneca AB, Sodertalje, Sweden) 18 mg was injected into subarachnoid space. The patient was immediately repositioned to supine position with the left lateral lift and a dermatome sensation blockade level of T6 with pinprick was achieved. Surgery commenced at 09:02 and her vital signs were stable with BP 115/72mmhg, HR 83 bpm, SPO2 99% and RR 21 breaths/min. No discomfort was complained during the surgery. A healthy baby was delivered uneventfully by caesarean section at 09:06. Apgar Scores were 8 and 8 at 1 min and 5 min respectively. All vital signs of the patient were stable and within normal limits from delivery to 09:20. The patient’s BP declined rapidly to 83/52 mmHg with HR 75 bpm at 9:20. No complaint was noted at that time. A dose of 2 mg methoxamine was administered intravenously. Two minutes later BP was 73/42 mmHg with HR 70 bpm. A second dose of 2 mg methoxamine was given. However, BP still showed a tendency of decreasing. A total of 6 mg methoxamine was used discontinuously in 8 min. The hemodynamic stability was still not achieved and the lowest BP dropped to 65/35 mmHg. Dopamine 2 mg was used at 09:31 and BP was 82/43 mmHg with HR78bpm at that time. There was no significant improvement in circulation. A total of 8 mg dopamine was administered within 8 min. Operation was completed with BP 96/55 mmHg, HR 82 bpm, SPO2 99% at 09:35. Estimated blood loss and urine volume were 200 ml and 200 ml respectively. A total of 800 ml crystalloid fluid was given. During the whole operation period, the patient did not complain any discomfort and was admitted to ICU at 09:45. The first BP measured in the ICU was 105/67mmhg. Morphine 1 mg was given via the epidural catheter. Ibuprofen (600 mg) and acetaminophen (650 mg) were orally used every 6 h for 2 days after surgery. The patient subsequently experienced an uneventful postpartum course without any neurological deterioration nor new-onset neurological sequelae. There was also no significant improvement in the pre-existing pathological features and clinical symptoms.

## Discussion and conclusions

This patient had atlantoaxial subluxation due to a congenital malformation of the cervical spine, which led to axial dentate protruding into the skull. ACM caused by CAAD is rare in clinical practice. Syringomyelia from C2 to C9 further complicated the situation. The formation of syringes may contribute to the craniospinal pressure gradient of CSF which was developed by the compression of the downward dislocation of cerebellar tonsils [4]. Patients with ACM-1 are more likely to have the syrinx in the cervicothoracic region. Few patients suffer syringes which only involve the lumbar spine region. The syringomyelia associated with ACM is commonly the communicating syringomyelia which means a direct communication between the syrinx and the CSF-filled cerebral ventricles or subarachnoid space [5]. The autonomic nervous system is not uncommonly involved, thus some certain symptoms of autonomic dysfunction may occur. It’s worth noting that accidental dural puncture with larger size needles could lead to the decreased pressure of CSF which can enlarge this communicating syrinx, thus exacerbating preexisting neurological symptoms.

The preferable choice of delivery mode (cesarean section VS vaginal delivery) and anesthesia (general anesthesia VS neuraxial technique) for patients with CAAD and ACM-1 has always been a very confusing issue in clinical practice. The Valsalva manoeuvre in vaginal delivery or bolus injections into the epidural or subarachnoid space may cause significant fluctuations in CSF pressure. The fluctuation tend to increase the degree of cerebellar herniation, enlarge the syrinx and make the CSF in the syrinx hit against the local nerve fiber, thereby worsen neurological complications. Severely restricted cervical movement and limited mouth opening resulting from the coexisting CAAD and deglutition disorder should also be highlighted. All these factors put our patient at the higher risk of difficult airway and aspiration than patients only with ACM-1 and syringomyelia when general anesthesia was chosen. Pathophysiological changes of coexisting severe preeclampsia in this case further complicated anesthesia management. The pathologic changes described above put anesthesia selection and intraoperative management into a giant dilemma.

Different medical institutions have their preferable anesthetic technique for patients with CAAD and ACM accompanied by syringomyelia. There are currently no firm guidelines to reference. Ghaly et al. reported a parturient with ACM- I and syringomyelia who continuously suffered kyphosis of the neck and limited neck mobility. The anesthesiologist performed general anesthesia with awake intubation for cesarean section [6]. However, some literatures also reported the successful management of neuraxial anesthesia for cesarean section in women with ACM-1. Single shot spinal anesthesia was successfully performed in patients affected by asymptomatic ACM − 1 without syringomyelia [7, 8]. Combined spinal-epidural technique was reported for labor analgesia in a parturient without syringomyelia and the preexisting ACM-related neurological symptoms did not become worse [9]. A multicenter retrospective study showed that more institutional preference in anesthesia was neuraxial procedure in parturients with ACM-1 and no case experienced exacerbation of pre-existing neurological symptoms. The incidence of accidental dural puncture was not different from that of the general maternal population, but the relative risk of PDPH may be higher in patients with syringomyelia [10].

A study has shown that patients with CAAD are more likely to develop respiratory impairment after general anesthesia. It is thought to be due to the site of the compression. The spinal cord from C1 to C2 is compressed. The neural tracts related to voluntary control of respiration (corticospinal tracts) lie in the lateral and ventral columns of the spinal cord. However, in patients with CAAD, the compression of the spinal cord by the odontoid process occurs near the midline. This compression would be likely to affect the anterior corticospinal tracts and the cell bodies concerned with respiratory muscle [11]. Owning to the neuroanatomic characteristics of the respiratory muscles mentioned above, the respiratory function in patients with CAAD is more likely to be impaired after general anesthesia. Therefore, respiration must be closely monitored and supported if necessary in this population. This was also the important reason why we tried to avoid general anesthesia as much as possible.

As outlined above, it should also be concerned that awake endotracheal intubation may induce cough which would lead to increased intracranial pressure. Furthermore, awake intubation is rarely used in our daily practice of anesthesia. Comparing with the limited proficiency of awake intubation, anesthetists in our institution have well skilled neuraxial technique. It would be very unlikely to risk accidental dural puncture. Concerned with available technical equipments and optimized performance of anesthesiologist in our hospital, combined spinal and epidural anesthesia would be supposed to maximize the benefit/risk ratio of this presented case.

Our patient experienced a widely fluctuating arterial pressure after delivery and showed less sensitivity to the commonly used vasoconstrictors. The progressive dysphagia of this patient indicated that the IXth and Xth cranial nerve had been disturbed. A study in patients with syringomyelia showed that vagal cardiovascular reflexes were impaired in patients with clinical evidence of involvement of the Xth cranial nerve. Even the symptoms of autonomic dysfunction are absent, cardiovascular reflexes in patients with syringomyelia may also be impaired [12]. Therefore, autonomic neuropathy was likely to occur in our patient and had been postulated to cause the insensitivity to vasoactive drugs. Though prior identification of autonomic involvement was important to avoid the great change of hemodynamics, the assessment of the autonomic nervous system was overlooked in this patient. There was one more explanation that the decreased intra-abdominal after delivery made the CSF pressure decline further in the lumbar spinal. This increased the craniospinal pressure gradient which may aggravate the herniation of medulla oblongata. The medulla oblongata was correspondingly displaced further to the caudal side and bore more pressure. The increased pressure on the medullary vasular center may attribute to the fluctuation of circulation and cause the insensitivity to peripheral action of vasoconstrictors.

In conclusion, the most important goal is to avoid further increasing the pressure gradient of CSF between the brain and spine during the anesthetic management of patients with CAAD and ACM-1 accompanied by syringomyelia. The key points on this issue include (1) slow injection of bolus through the spinal or epidural needle preventing undue CSF pressure fluctuation; (2) the anesthesia procedure performed by an experienced and trained anesthesiologist avoiding accidental dural puncture as well as multiple needle attempts; (3) trying best to stabilize the cervical spine during intubation; (4) the avoidance of cough and bucking during general anesthesia. No matter which type of anesthesia was chosen in our case, risks should always be considered. A multidisciplinary technique was needed to weigh risks and benefits carefully. Furthermore, anesthesiologists should make an individual anesthetic plan based on the available clinical anesthesia equipments and their experience on professional techniques.

## Data Availability

Not applicable.

## Abbreviations

CAAD: Congenital atlantoaxial dislocation
ACM: Arnold Chiari malformations
(ACM-I): type I Arnold Chiari malformations
MRI: Magnetic resonance imaging
CT: Computed Tomography
BP: Blood pressure
HR: Heart rate
RR: Respiratory rate
ICU: Intensive care unit
CSF: Cerebrospinal fluid
PDPH: Post-dural puncture headache

## References

[1] Salunke P, Sahoo SK, Deepak AN (2016). Redefining congenital atlantoaxial dislocation: objective assessment in each plane before and after operation. World Neurosurg.

[2] Dyste GN, Menezes AH, Van Gilder JC (1989). Symptomatic Chiari malformations. J Neurosurg.

[3] Meadows J, Kraut M, Guarnieri M (2000). Asymptomatic chiari type I malformations identified on magnetic resonance imaging. J Neurosurg.

[4] Oldfield EH, Muraszko K, Shawker TH (1994). Pathophysiology of syringomyelia associated with chiari I malformation of the cerebellar tonsils. Implications for diagnosis and treatment. J Neurosurg.

[5] Walton Sir J (1985). Brain’s diseases of the nervous system.

[6] Ghaly RF, Candido KD, Sauer R (2012). Anesthetic management during cesarean section in a woman with residual Arnold-Chiari malformation type I, cervical kyphosis, and syringomyelia. Surg Neurol Int.

[7] EMMEZ G, ARIK E, ÖZDEMİR Ç (2018). Management of spinal anesthesia for parturient with Arnold Chiari malformation type-1. Anestezi Dergisi.

[8] Gunaydin B, Emmeze G, Das O (2012). Caesarean delivery for twin pregnancy: spinal anesthesia for asymptomatic type 1 Arnold Chiari malformation. Anestezjologia i Ratownictwo.

[9] Choi CK, Tyagaraj K (2013). Combined spinal-epidural analgesia for laboring parturient with Arnold-Chiari type I malformation: a case report and a review of the literature. Case Rep Anesthesiol.

[10] Gruffi TR, Peralta FM, Thakkar MS (2019). Anesthetic management of parturients with Arnold Chiari malformation-I: a multicenter retrospective study. Int J Obstet Anesth.

[11] Reddy KR, Rao GS, Devi BI (2009). Pulmonary function after surgery for congenital atlantoaxial dislocation: a comparison with surgery for compressive cervical myelopathy and craniotomy. J Neurosurg Anesthesiol.

[12] Nogués MA, Newman PK, Male VJ (1982). Cardiovascular reflexes in syringomyelia. Brain.

